# Evidence gap on antihyperglycemic pharmacotherapy in frail older adults: A systematic review

**DOI:** 10.1007/s00391-020-01724-3

**Published:** 2020-04-17

**Authors:** Claudia Bollig, Gabriel Torbahn, Jürgen Bauer, Simone Brefka, Dhayana Dallmeier, Michael Denkinger, Annette Eidam, Stefan Klöppel, Andrej Zeyfang, Sebastian Voigt-Radloff, MedQoL-Group (Medication and Quality of Life in frail older persons)

**Affiliations:** 1 Institute for Evidence in Medicine, Medical Center—University of Freiburg, Faculty of Medicine, University of Freiburg, Hugstetter Str. 49, 79106 Freiburg, Germany; 2 Cochrane Germany, Cochrane Germany Foundation, Freiburg, Germany; 3 Institute for Biomedicine of Aging, Friedrich-Alexander-Universität Erlangen-Nürnberg, Nuremberg, Germany; 4 Center for Geriatric Medicine, University of Heidelberg and Agaplesion Bethanien Hospital, Heidelberg, Germany; 5 Agaplesion Bethesda Clinic, Geriatric Research Unit Ulm University, Ulm, Germany; 6 University Hospital of Old Age Psychiatry and Psychotherapy, University of Bern, Bern, Switzerland; 7 Department of Epidemiology, University of Ulm, Ulm, Germany; 8 Center for Geriatric Medicine and Gerontology Freiburg, Medical Center Faculty of Medicine, University of Freiburg, Freiburg, Germany

**Keywords:** Aged, Frailty, Diabetes mellitus, Drug therapy, Systematic review, Ältere Menschen, Ältere gebrechliche Menschen, Diabetes mellitus, Medikamentöse Therapie, Systematisches Review

## Abstract

**Background:**

Although antihyperglycemic pharmacotherapy in frail older adults with type 2 diabetes mellitus (T2DM) is challenging, recommendations from international guidelines are mainly based on indirect evidence from trials not including frail participants.

**Objective:**

This systematic review investigated the effectiveness and safety of pharmacotherapy in frail older adults with T2DM.

**Material and methods:**

Randomized (RCT) and non-randomized prospective clinical trials (non-RCT) were searched in three electronic databases (Medline, Embase, Central) up to October 2018. Trials in older adults with T2DM who were assessed as significantly or severely impaired by defined cut-off scores of assessment instruments on frailty, activities of daily living or physical functional impairment were included.

**Results:**

Two reviewers independently screened 17,391 references for inclusion and assessed risk of bias with ROBINS‑I. Five non-RCTs and no RCT were identified. Treatment of T2DM without insulin compared to insulin could be associated with increased improvement in cardiac functions in patients with cardiac resynchronization therapy and with decreased falls in frail older women. While better glycemic control with low variability and low HbA1c (hemoglobin A1c) values (<7%) was associated with better maintenance of physical function in community-dwelling older persons, higher HbA1c values (8–8.9%) were associated with a reduction in the composite outcome of death or functional decline in community-dwelling diabetic older adults with need for skilled assistance. Due to serious risk of bias in all studies, results should be considered with caution.

**Conclusion:**

Well-designed, large-scale RCTs including this important group of patients are required to assess the effectiveness and safety of pharmacotherapy and HbA1c targets.

**Electronic supplementary material:**

The online version of this article (10.1007/s00391-020-01724-3) contains supplementary material, which is available to authorized users.

## Abstract (de)

**Hintergrund:**

Auch wenn die antihyperglykämische Pharmakotherapie von älteren, gebrechlichen Patienten mit Typ-2-Diabetes anspruchsvoll ist, basieren die Empfehlungen internationaler Leitlinien hauptsächlich auf indirekter Evidenz aus Studien, die gebrechliche Patienten nicht einschließen.

**Ziel der Arbeit:**

Dieser systematische Review untersuchte die Wirksamkeit und Sicherheit medikamentöser Therapien bei gebrechlichen, älteren Patienten mit Typ-2-Diabetes.

**Material und Methoden:**

Bis Oktober 2018 wurden systematische Literatursuchen in drei Datenbanken (Medline, Embase, Central) nach randomisierten (RCTs) und prospektiven nichtrandomisierten kontrollierten Studien (Non-RCTs) durchgeführt. Studien bei älteren Erwachsenen mit Typ-2-Diabetes, die durch vorab definierte Schwellenwerte hinsichtlich Gebrechlichkeit, Aktivitäten des täglichen Lebens oder anderer Instrumente zur Messung der körperlichen Gebrechlichkeit als signifikant oder schwerwiegend funktionell eingeschränkt beurteilt worden waren, wurden eingeschlossen.

**Ergebnisse:**

Zwei Review-Autoren untersuchten unabhängig voneinander die Einschlussfähigkeit von 17.391 Datenbankeinträgen. Es wurden fünf Non-RCTs und keine RCT identifiziert. Die Therapie von Typ-2-Diabetes ohne Insulin könnte im Vergleich zu einer Therapie mit Insulin mit stärkeren Verbesserungen der Herzfunktion bei Patienten mit kardialer Resynchronisation und mit weniger Stürzen bei gebrechlichen älteren Frauen assoziiert sein. In einer Studie war eine bessere glykämische Kontrolle, definiert durch weniger schwankende und niedrigere HbA1c-Werte (<7 %) mit einer besseren Erhaltung der körperlichen Funktion bei zu Hause lebenden gebrechlichen Älteren mit Typ-2-Diabetes assoziiert. In einer anderen Studie waren höhere HbA1c-Werte (8–8,9 %) mit einer Reduktion des zusammengesetzten Endpunkts aus Mortalität oder funktioneller Beeinträchtigung bei älteren, zu Hause lebenden pflegebedürftigen Erwachsenen mit Typ-2-Diabetes assoziiert. Aufgrund eines schwerwiegenden Risikos für Bias in allen Studien sollten die Ergebnisse mit Vorsicht betrachtet werden.

**Diskussion:**

Gut konzipierte, große randomisierte kontrollierte Studien, die gebrechliche, ältere Patienten explizit adressieren sind notwendig, um die Wirksamkeit und Sicherheit der medikamentösen Therapie und der HbA1c-Ziele einzuschätzen.

## Introduction

Since 1980 the global prevalence of diabetes mellitus (DM) has nearly doubled up to 422 million people worldwide in 2014 and is estimated to redouble in the next 20 years [44]. In high-income countries, DM prevalence was 3 times higher among 65–69 year old patients compared to low income countries and peaked in the 75–79 years age group (22%) [6]. Late complications of DM, such as myocardial infarction, stroke, renal failure, peripheral arterial occlusive disease, diabetic retinopathy and polyneuropathies, negatively impact the quality of life and daily functioning of patients, and represent major costs within healthcare systems [45]. In addition, hypoglycemia has been associated with higher mortality and increased risks for falls and fall-related injuries [23].

In the older diabetic population, type 2 diabetes mellitus (T2DM) has a prevalence of up to 90% [12]. Its antihyperglycemic pharmacotherapy is challenging [35], particularly among frail older adults with impaired physical functions [36]. Diabetes mellitus may increase the risk for frailty [10] and they both share several pathophysiological mechanisms, e.g. chronic low-grade inflammation, insulin resistance and sarcopenia [2, 29], the latter being a major contributing factor to pathophysiology of frailty.

Several national and international medical societies have published guidelines for both T2DM [1, 13, 14, 25, 37, 39] and frail older persons with T2DM [22]. However, recommendations are mainly based on indirect evidence from trials not including frail participants or excluding older adults at all [14, 21].

This research group on medication and quality of life in frail older persons (MedQoL-Group) conducted four extended systematic reviews on drug treatment in hypertension [28], depression, dementia and T2DM. We included trials with older persons who can be characterized as frail in terms of impaired physical functioning, even if the investigators of the primary studies did not explicitly consider the concept of frailty when planning their studies. This article reports a systematic review of randomized (RCT) and non-randomized prospective clinical trials (non-RCT) intended to determine the effectiveness and safety of pharmacotherapy for frail older persons with T2DM characterized as functionally significantly or severely impaired.

## Material and methods

This systematic review is reported according to the PRISMA guidelines [20] and has been specified in a protocol registered on PROSPERO (CRD42018108997, https://www.crd.york.ac.uk/prospero).

### Search and study eligibility

The databases MEDLINE (via Ovid), Embase (via Ovid) and Cochrane Central Register of Trials (CENTRAL) were searched for randomized and non-randomized prospective clinical trials comparing antihyperglycemic pharmacotherapy versus placebo or other antihyperglycemic medication or comparing different HbA1c targets in older persons with DM (see table S1 and table S2 for Medline search strategies). In addition, reference lists of included studies were screened. Detailed search methods are described in S3. Titles/abstracts and full texts were assessed for eligibility by two reviewers (CB, GT), independently. Conflicts were solved by discussion or a third reviewer (SVR).

RCTs and non-RCTs with control group design reporting on persons aged 65 years or older (or mean age ≥70 years) with T2DM and significant or severe impairment of physical function were included. We assessed functional impairment according to a compilation of predefined thresholds in 51 established indices and scores [4]. For preparation of this compilation, we limited our scope of interest to assessments of frailty and, as proxy indicators, instruments evaluating activities of daily living (ADL) or physical functioning or impairment. With regard to the latter we excluded assessments of cognitive functioning. For each identified instrument, we defined cut-off scores for the categories (1) functionally independent, (2) functionally slightly impaired, (3) functionally significantly impaired or partially dependent and (4) functionally severely impaired or disabled or dependent. In our review, we only considered at least significantly impaired populations.

### Data extraction and risk of bias assessment

One author extracted data from each study and another checked data for accuracy and completeness. The two reviewers worked independently on risk of bias assessment. Conflicts were again solved by discussion or consulting the third reviewer. Data extraction was carried out in piloted data forms. The reviewers used the risk of bias in nonrandomized studies of interventions (ROBINS‑I) tool to assess the risk of bias of included prospective cohort studies [40].

## Results

**Fig. 1:**
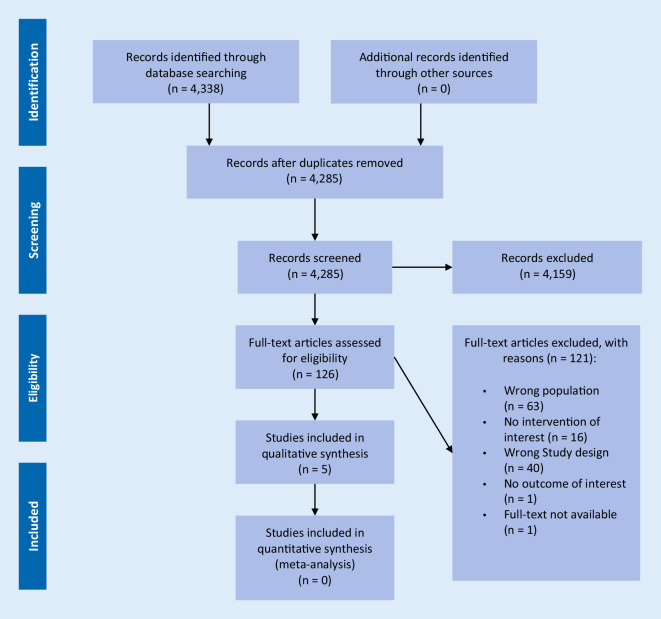
Flowchart: selection of non-RCTs [26] (For more information visit www.prisma-statement.org)

We included five non-RCTs and no RCT involving 1220 older persons with DM. The flow of non-RCT and RCT inclusion is presented in the PRISMA flowcharts (Fig. 1﻿ and S1 Fig).

Characteristics of included studies are mentioned in Table 1. Mean ages ranged from 73.8 to 81.7 years, follow-up periods from 1 to 7 years. Studies were performed from 1986 to 2012. Three trials were supported by public grant [33, 43, 46]. One study reported to have no conflict of interest [32]. One study did not report on conflict of interests [30]. All studies were assessed as serious risk of bias, mainly due to important confounders not considered in the analyses.

**Table 1:** Characteristics of included studies

Study	Setting	Age years, mean (SD)	Female percent	Functional status at baseline mean (SD)	Comparison	Outcomes reported	Risk of Bias
Schwartz 2002 [33] *n* = 629	Community-dwelling	73.8 (5.0)^a^	100	Any difficulty in IADL: 59%^a^ Grip strength: 17.9 kg (4.7)^a^ Quadriceps strength: 55.4 kg (27.5)^a^ 5‑chair test: 13.8 s (5.2)^a^ Poor tandem stand: 60%^a^ 2‑m tandem walk: 19.1 s (8.8)^a^ Walking speed: 0.8 m/s (0.2)^a^	Insulin vs. no insulin	Falls	Serious
Pham 2003 [30] *n* = 73	Nursing and residential homes	76.0 (7.9)	63^a^	Katz ADL: 70% dependent	Insulin vs. oral drugs vs. no pharmacological antidiabetic treatment HbA1c (%): <6.5% vs. 6.5–8% vs. >8%	Hypoglycemia Depression ADL Mortality	Serious
Wang 2011 [43] *n* = 119	Community-dwelling	76.3 (3.4)	55	SPPB: 7.6 (3.4)	HbA1c: poorer vs. better control class	Lower extremity function	Serious
Yau 2012 [46] *n* = 367	Community-dwelling with need for skilled help	80 (9)	67	ADL-score ≤8: 66%	HbA1c (%): <7.0% vs. 7.0–7.9% vs. 8.0–8.9% vs. ≥9.0%	Functional decline/death	Serious
Sardu 2014 [32] *n* = 32	Hospital patients with cardiac resynchronization therapy	81.7 (6.3)	41	6‑min walk: 249 m (50.7)	Insulin vs. no insulin	6‑min walk HbA1c NYHA class	Serious

*ADL-score* consists of five activities of daily living (bathing, dressing, toileting, transferring and eating), ranges from 10 (independent) to 0 (completely dependent), *HbA1c* hemoglobin A1c, *IADL* Instrumental activities of daily living, *Katz ADL* consists of six activities of daily living (bathing, dressing, toileting, transfer, continence, eating) ranges from 0 (independent) to 12 (maximal level of dependency), *kg* kilogram, *m* meter, *min* minutes, *NYHA* New York Heart Association, *s* seconds, *SPPB* short physical performance battery

^a^Calculated by review authors

## Insulin versus oral antihyperglycemics versus no pharmacological antidiabetic treatment

Pham et al. compared 73 diabetic nursing home residents older than 60 years regarding patterns of effective management, DM control and functional dependency [30]. This observational study was conducted in two nursing and two residential homes in France. Items of DM control and follow-up were prospectively recorded. Functional dependency was assessed using the Katz index of activities of daily living [17]. Katz scores were not reported on detail at baseline, but only a minor subsample (*n* = 17) were classified as functionally independent. Change in DM management from baseline to the 18 months follow-up occurred in participants receiving insulin from 26 to 27, oral antihyperglycemics from 29 to 24, and no treatment or diet alone from 18 to 22. Hypoglycemia occurred in 24 patients: 18 of 27 (67%) on insulin and 6 of 24 (25%) on oral antihyperglycemics. At the end of the study 41 subjects had depression: 15 of 27 (56%) on insulin, 14 of 24 (58%) on oral antihyperglycemics and 12 of 22 (55%) without treatment or diet alone. Associations between DM management and functional dependency were not reported for any subgroup.

## Different HbA1c values

Pham et al. furthermore classified the 73 diabetic nursing home residents according to the lowest HbA1c value: 19 participants had values lower than 6.5%; 20 between 6.5% and 8%; 15 higher than 8% [30]. In 19 subjects, HbA1c was never measured during the study period. At the end of the 18-months follow-up, 7 (36.8%), 5 (25%) and 4 (26.7%) participants had died, respectively. In univariate ANOVA analysis and χ^2^-test no relationships were found between HbA1c range and changes in the Katz index or mortality rate.

Wang and Hazuda evaluated the impact of glycemic control on the maintenance of lower-extremity physical function in 119 diabetic participants aged 71 years or older [43]. Using a latent growth mixture modelling, poor glycemic control class was defined by means of HbA1c > 7% and higher intrasubject variability, better control by means of HbA1c < 7% and lower intra-subject-variability. The comparison of adjusted correlations in the change from baseline scores of the Short Physical Performance Battery (SPPB) at 18 and 36 months revealed that improved glycemic control was associated with a better maintenance of physical function.

Yau et al. evaluated if HbA1c levels predicted functional decline or death in 367 functional severely impaired community-dwelling older adults who were unable to live independently [46]. Functionality was assessed based on five ADLs (bathing, dressing, toileting, transferring and eating) scoring between 0 (completely dependent) and 2 points (independent) in each activity. Two-thirds of participants had a baseline ADL score of 8 or less. Mortality, functional decline plus the composite outcome of mortality and functional decline were reported after 6, 12 and 24 months. After accounting for confounders age, sex, race or ethnicity, time enrolled in the study, baseline activities of daily living, comorbidities (cancer, congestive heart failure, chronic obstructive lung disease, renal disease, dialysis) and medications (none, oral medication, insulin), patients with HbA1c levels of 8–8.9% had the lowest and levels of <7% the highest risk of death, functional decline and composite outcome at 2 years. Only the result for the composite outcome in the 8.0–8.9% group reached statistical significance. Stratification of analysis according to antidiabetic medications (oral antihyperglycemic medications only vs. any insulin) showed similar results in both groups.

## Insulin versus no insulin

Schwartz et al. determined risk factors and the risk of falls in 530 insulin treated and 99 non-insulin treated diabetic women aged 65 years or older [33]. At baseline, there were significant differences in characteristics of insulin treated and non-insulin treated participants, e.g. 71% of insulin treated versus 57% of non-insulin treated women had difficulties in instrumental activities of daily living (walking two or three blocks, climbing 10 steps, preparing own meals, doing heavy housework and doing own shopping). Time since diagnosis of diabetes also differed (17.4 ± 11.2 versus 11.3 ± 9.2 years). Functional measures at baseline were grip strength, quadriceps strength, walking speed, 5 chair test, tandem stand and tandem walk. Within the 2‑year follow-up, the incidence of falls per person-year between the insulin and non-insulin group significantly differed in the total study sample (1.12 vs. 0.85) and in age subgroups (70–74 years: 1.26 vs. 0.56; 80–84 years: 1.31 vs. 0.89). Significantly more women taking insulin fell more than once a year (35.4%) compared to women receiving no insulin (25.7%). There was no significant difference in persons who fell more than twice a year (10.6% vs. 15.2%). Tandem walk accounted for 23% of the association between non-insulin-treated DM and falling, which was the largest contributing factor among all risk factors. Together with poor performance on tandem stand, both measures accounted for 30% of the association between non-insulin-treated DM and falling and 26% of the association between insulin-treated DM and falling.

Sardu et al. determined the effects of insulin (*n* = 12) versus no insulin therapy (*n* = 18) in diabetic patients with heart failure older than 75 years and receiving a cardiac resynchronization therapy implant [32]. The 6‑min walk improved from baseline to 12 months in the insulin group from 243.2 (SD 52.2) to 259.6 (53.7) m and in the non-insulin group from 254.7 (55.1) to 271.3 (56.4) m. Changes in 6‑min walk differed not significantly between groups. Mean New York Heart Association (NYHA) functional class improved in the insulin group from 3.38 (1.1) to 2.8 (0.7), but significantly more in the non-insulin group from 3.21 (1.0) to 2.05 (0.6). HbA1c values remained stable in both groups compared to baseline. Quality of life was not reported.

## Discussion

There is very low evidence that treatment of T2DM without insulin compared to insulin therapy might be associated with improvement in cardiac functions, in older diabetic patients with cardiac resynchronization therapy [32] and with decreased falls, in frail older women [33]; however, subjects might have received insulin due to severe diabetes of longer duration, which may explain adverse outcomes. While better glycemic control with low variability and low HbA1c values (<7%) was associated with better maintenance of physical function in community-dwelling older persons [43], higher HbA1c values (8.0–8.9%) were associated with a reduction in the composite outcome of death or functional decline in community-dwelling diabetic older adults with need for skilled assistance (classified as nursing home eligible [46]).

## Strengths and limitations

Strengths of this study were (1) the expanded search (2) the use of up to date and specific tools to assess the risk of bias, and (3) the independent screening and risk of bias assessment by two reviewers. Although we specified assessments with cut-off scores of frailty indicators a priori and dependency in ADL has been validated as frailty proxy [8], the main limitation of our systematic review was the use of proxy indicators and the retrospective identification of the frail population in eligible trials.

Based on the included studies, all with high risk of bias, and the indirect evidence from excluded studies in the research field (S4), we cautiously interpreted that a critical assessment of the need for insulin and its conservative use as well as multimodal strategies to set and reach individual moderate HbA1c targets [47] might have beneficial effects in diabetic frail older adults. Considering the findings that both low and high HbA1c values were associated with increased mortality and cardiac events [7], we recommend to follow current guidelines on T2DM in older persons proposing a defined target range of HbA1c levels (7.5–8.5%) [1, 3]; however, hypothesized positive effects of conservative insulin use and defined HbA1c target ranges are to be evaluated in future rigorous research.

## Conclusion

### Implications for practice

The very low evidence from the included trials and the additional discussion of indirect evidence from the excluded studies suggest an essential evidence gap so that firm recommendations for antihyperglycemic pharmacotherapy in frail older adults with physical functional impairment cannot be drawn.

### Implications for research

Well designed, large-scale RCTs, including not the same team replications [15], are required to establish the effectiveness of implementing internationally established guidelines, based on the most recent position papers managing frailty in DM [38, 41]. To address the well-known challenges in research with frail older persons [34], we suggest a prospective network meta-analysis [42] as methodological framework as well as coordinated practice-based research networks [11, 27] and patient representatives as stakeholder partners to ensure rigorous scientific methods, sufficient recruitment and a patient-centered and practice-oriented study plan [9]. An evidence-based, updated study plan involving networking and methodological expertise [5, 18] of ongoing or recently published RCTs in the field [19, 24, 31] should be established, therefore avoiding waste in research [16].

## Caption Electronic Supplementary Material

S1 Table. Search strategy RCTs (Medline via Ovid).

S2 Table. Search strategy non-RCTs (Medline via Ovid).

S1 Fig. Flowchart: Selection of RCTs.

S3. Search methods.

S4. Indirect evidence from excluded studies
