# Supplementary material for: Evidence gap on antihyperglycemic pharmacotherapy in frail older adults: A systematic review
Source: Z Gerontol Geriatr. 2020 Apr 17;54(3):278–84. doi: 10.1007/s00391-020-01724-3 (PMC8096761; doi:10.1007/s00391-020-01724-3)
Supplement: Supplementary file 2 — S2 Table. Search strategy non-RCTs (Medline via Ovid). [file 391_2020_1724_MOESM2_ESM.pdf]

Search strategy non-RCTs

Search date: 10/24/2018

Ovid MEDLINE(R) Epub Ahead of Print October 23, 2018

In-Process & Other Non-Indexed Citations October 23 2018

Ovid MEDLINE(R) Daily Update October 23, 2018

Ovid MEDLINE(R) 1946 to October Week 2 2018

1. exp Diabetes Mellitus/
2. (Type* adj3 ("2" or "II" or two*) adj3 (diabete* or diabetic*)).ti,ab,kf.
3. ((Maturit* or adult* or slow*) adj3 onset* adj3 (diabete* or diabetic*)).ti,ab,kf.
4. ((Ketosis-resistant* or stable*) adj3 (diabete* or diabetic*)).ti,ab,kf.
5. ((Non-insulin* or Noninsulin*) adj3 depend* adj3 (diabete* or diabetic*)).ti,ab,kf.
6. NIDDM.ti,ab,kf.
7. 1 or 2 or 3 or 4 or 5 or 6
8. drug therapy/ or exp drug administration routes/ or exp drug administration schedule/ or exp drug delivery systems/ or drug dosage calculations/ or exp drug prescriptions/ or exp drug therapy, combination/ or drug therapy, computer-assisted/ or inappropriate prescribing/ or exp medication errors/ or exp polypharmacy/ or self administration/ or self medication/
9. drug therap*.ti,ab,kf.
10. drug therapy.fs.
11. (pharmacotherap* or pharmaco-therap*).ti,ab,kf.
12. drugs.ti,ab,kf.
13. medication.ti,ab,kf.
14. or/8-13
15. Dipeptidyl-Peptidase IV Inhibitors/
16. (Dipeptidyl* adj2 Peptidase* adj2 ("4" or "iv") adj Inhibitor*).ti,ab,kf,nm.
17. (Dipeptidylpeptidase* adj2 ("4" or "iv") adj Inhibitor*).ti,ab,kf,nm.
18. (DPP* adj2 ("4" or "iv")).ti,ab,kf,nm.
19. Gliptin*.ti,ab,kf,nm.
20. (Sitagliptin* or Januvia*).ti,ab,kf,nm.
21. (Vildagliptin* or Galvus*).ti,ab,kf,nm.
22. (Linagliptin* or Trajenta*).ti,ab,kf,nm.
23. (Saxagliptin* or Onglyza*).ti,ab,kf,nm.
24. (Alogliptin* or Nesina*).ti,ab,kf,nm.
25. or/15-24
26. exp Glucagon-Like Peptide 1/
27. (Glucagon* adj Like adj Peptide adj "1").ti,ab,kf,nm.
28. (GLP* adj "1").ti,ab,kf,nm.
29. (Exenatid* or Byetta* or Bydureon*).ti,ab,kf,nm.
30. (Liraglutid* or Victoza* or Dulaglutid* or Trulicity* or Albiglutid* or Tanzeum*).ti,ab,kf,nm.

31. (Lixisenatid* or Lyxumia* or Adlyxin*).ti,ab,kf,nm.
32. or/26-31
33. Thiazolidinediones/
34. (Thiazolidinedion* or Glitazon*).ti,ab,kf,nm.
35. (Pioglitazon* or Actos*).ti,ab,kf,nm.
36. or/33-35
37. exp Sulfonylurea Compounds/
38. (Sulfonylurea* or Sulphonylurea*).ti,ab,kf,nm.
39. (Gl#benclamid* or Glyburid*).ti,ab,kf,nm.
40. (Glimepirid* or Amaryl*).ti,ab,kf,nm.
41. (Gl#clazid* or Diamicron*).ti,ab,kf,nm.
42. (Gliquidon* or Glurenor*).ti,ab,kf,nm.
43. or/37-42
44. exp Metformin/
45. (Metformin* or Glucophage*).ti,ab,kf,nm.
46. (Competact* or Janumet* or Eucreas*).ti,ab,kf,nm.
47. Biguanides/
48. Biguanid*.ti,ab,kf,nm.
49. or/44-48
50. exp Trisaccharides/
51. (Acarbos* or Glucobay*).ti,ab,kf,nm.
52. (Miglitol* or Diastabol* or Glyset*).ti,ab,kf,nm.
53. or/50-52
54. exp Insulins/tu [Therapeutic Use]
55. exp Insulin/ad [Administration & Dosage]
56. Insulin Infusion Systems/
57. (Insulin* adj3 (treat* or therap* or administrat* or dos* or human* or analogue* or biphasic* or basal* or protamin* or isophan* or inject* or pen* or deliver* or device* or system* or pump* or syringe* or needle*)).ti,ab,kf,nm.
58. (Insulin* adj3 (Intermediate* or shortact* or short-act* or longact* or long-act* or ultralong* or ultra-long*)).ti,ab,kf,nm.
59. (Actrapid* or Humulin* or Insuman* or Hypurin*).ti,ab,kf,nm.
60. (Aspart* or Novorapid*).ti,ab,kf,nm.
61. (Glulisin* or Apidra*).ti,ab,kf,nm.
62. (Lispro* or Humalog*).ti,ab,kf,nm.
63. (Insulin* adj3 zinc* adj3 (suspension* or protamin*)).ti,ab,kf,nm.
64. (Detemir* or Levemir*).ti,ab,kf,nm.
65. (Glargin* or Lantus*).ti,ab,kf,nm.
66. (Degludec* or Tresiba*).ti,ab,kf,nm.
67. (Isophan* or Insulatard* or Humulin* or Insuman* or Novomix*).ti,ab,kf,nm.
68. or/54-67
69. Sodium-Glucose Transporter 2/
70. (Sodium* adj3 Glucose* adj3 Transporter* adj3 "2").ti,ab,kf,nm.

71. (Sodium* adj3 Glucose* adj3 (co-transporter* or cotransporter*) adj3 "2").ti,ab,kf,nm.
72. SGLT*.ti,ab,kf,nm.
73. Gliflozin*.ti,ab,kf,nm.
74. (Canagliflozin* or Dapagliflozin* or Empagliflozin*).ti,ab,kf,nm.
75. Glinid*.ti,ab,kf,nm.
76. (Meglitinid* or Nateglinid* or Repaglinid*).ti,ab,kf,nm.
77. or/69-76
78. exp hypoglycemic agents/
79. (hypoglyc* or antihyperglyc* or anti-hyperglyc* or antidiabetic* or anti-diabetic*).ti,ab,kf,nm.
80. 25 or 32 or 36 or 43 or 49 or 53 or 68 or 77 or 78 or 79
81. elder*.ti,ab,kf.
82. (community adj1 dwelling).ti,ab,kf.
83. geriatric.ti,ab,kf.
84. "mini-mental state".ti,ab,kf.
85. alzheimer*.ti,ab,kf.
86. mmse.ti,ab,kf.
87. caregiver*.ti,ab,kf.
88. falls.ti,ab,kf.
89. adl.ti,ab,kf.
90. Gds.ti,ab,kf.
91. Ag?ing.ti,ab,kf.
92. frail*.ti,ab,kf.
93. Mci.ti,ab,kf.
94. exp dementia/
95. dement*.ti,ab,kf.
96. (psychogeriatric* or psycho-geriatric*).ti,ab,kf.
97. "cognitive impairment".ti,ab,kf.
98. "postmenopausal women".ti,ab,kf.
99. comorbid*.ti,ab,kf.
100. Geriatric Assessment/
101. exp Nursing homes/
102. Frail Elderly/
103. exp Cognition Disorders/di [Diagnosis]
104. exp Cognition disorders/ep [epidemiology]
105. Homes for the Aged/
106. disability.ti,ab,kf.
107. disabled persons/ or persons with hearing impairments/ or visually impaired persons/
108. "functional decline".ti,ab,kf.
109. (gerontopsychiatry or geronto-psychiatry).ti,ab,kf.
110. "activities of daily living".ti,ab,kf.

111. exp "Activities of daily living"/
112. immobility.ti,ab,kf.
113. immobilization.ti,ab,kf.
114. or/81-113
115. exp cohort studies/
116. cohort$.tw.
117. controlled clinical trial.pt.
118. epidemiologic methods/
119. limit 118 to yr=1971-1988
120. or/115-117,119
121. 7 and 14
122. 80 or 121
123. 114 and 120 and 122
