# Supplementary material for: Evidence gap on antihyperglycemic pharmacotherapy in frail older adults: A systematic review
Source: Z Gerontol Geriatr. 2020 Apr 17;54(3):278–84. doi: 10.1007/s00391-020-01724-3 (PMC8096761; doi:10.1007/s00391-020-01724-3)
Supplement: Supplementary file 3 — S1 Fig. Flowchart: Selection of RCTs. [file 391_2020_1724_MOESM3_ESM.pdf]

PRISMA 2009 Flow Diagram

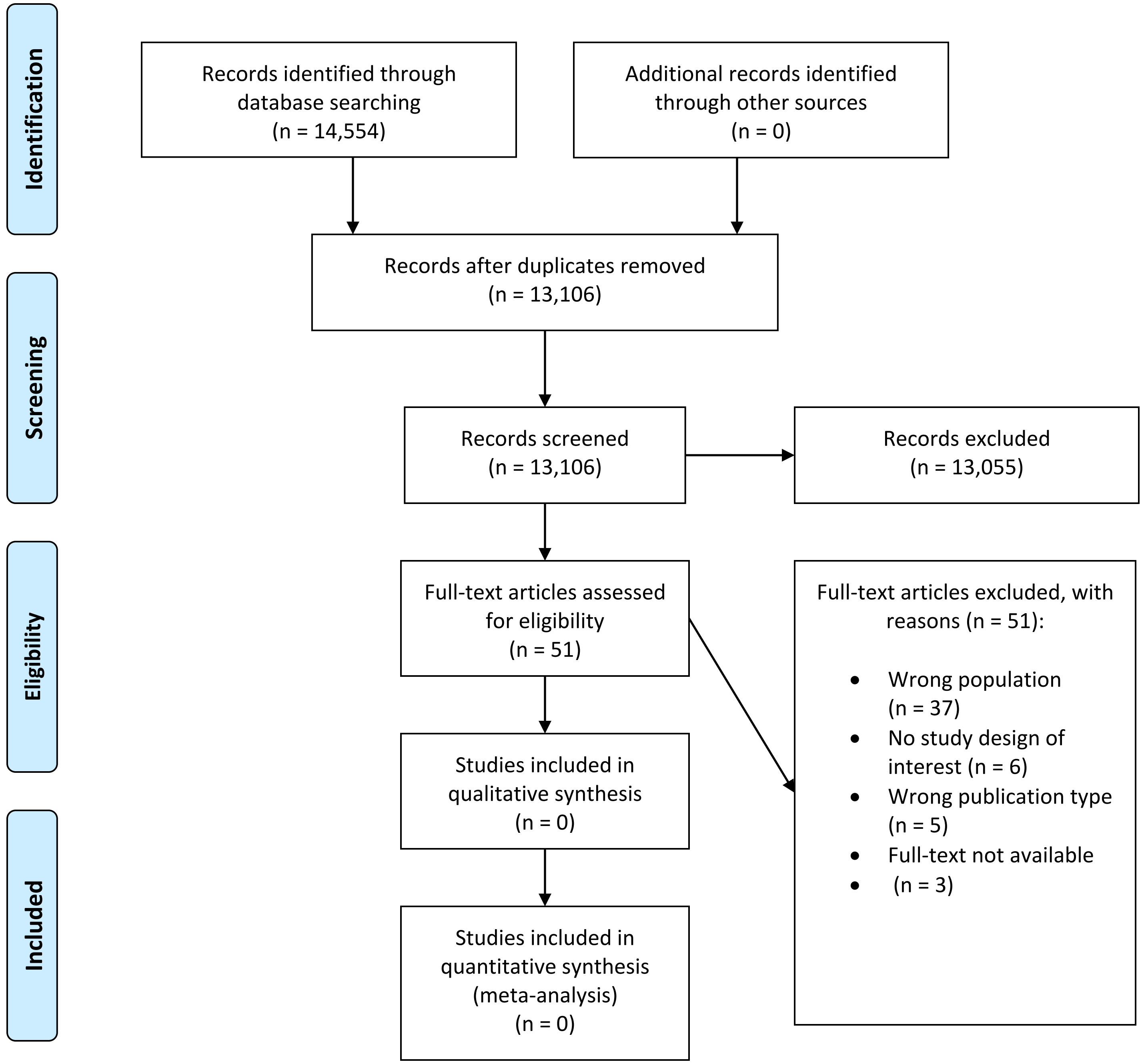

From: Moher D, Liberati A, Tetzlaff J, Altman DG, The PRISMA Group (2009). *P*referred *R*eporting *I*tems for *S*ystematic Reviews and *M*eta-*A*nalyses: The PRISMA Statement. PLoS Med 6(7): e1000097. doi:10.1371/journal.pmed1000097

For more information, visit www.prisma-statement.org.
