# Supplementary material for: Evidence gap on antihyperglycemic pharmacotherapy in frail older adults: A systematic review
Source: Z Gerontol Geriatr. 2020 Apr 17;54(3):278–84. doi: 10.1007/s00391-020-01724-3 (PMC8096761; doi:10.1007/s00391-020-01724-3)
Supplement: Supplementary file 4 — S3. Search methods. [file 391_2020_1724_MOESM4_ESM.pdf]

S3. Search methods

Established filters for geriatric patients [4] (supplemented with further search terms related to functionality) and trial designs were used to build two search strategies for RCTs (Cochrane search filter, sensitivity-maximizing version [2]) and non-RCTs (cohort study strategy, [1]). We combined these search strings with a comprehensive list of terms relating to T2DM and antihyperglycemic drugs based on a preparatory analysis of search strings of current systematic reviews and guidelines in the field [3]. Our search was not limited to language, publication date or geographic origin.

References:

1. Bmj Best Practice: Study design search filters. http://bestpractice. bmj.com/info/toolkit/learn-ebm/study-design-search-filters/ (last accessed on 5 Jun 2019)
2. Lefebvre C, Manheimer E, Glanville J (2011) Chapter 6: Searching for studies. In: Higgins JPT, Green S (eds) Cochrane Handbook for Systematic Reviews of Interventions Version 5.1.0 (updated March 2011). The Cochrane Collaboration
3. National Institute for Health and Care Excellence (2015) Type 2 diabetes in adults: management. (NICE guideline 28). www.nice.org.uk/guidance/ng28 (last accessed on 06 March 2017)
4. Van De Glind EM, Van Munster BC, Spijker R et al. (2012) Search filters to identify geriatric medicine in Medline. Journal of the American Medical Informatics Association : JAMIA 19:468-472
