# Supplementary material for: Evidence gap on antihyperglycemic pharmacotherapy in frail older adults: A systematic review
Source: Z Gerontol Geriatr. 2020 Apr 17;54(3):278–84. doi: 10.1007/s00391-020-01724-3 (PMC8096761; doi:10.1007/s00391-020-01724-3)
Supplement: Supplementary file 5 — S4. Indirect evidence from excluded studies [file 391_2020_1724_MOESM5_ESM.pdf]

S4. Indirect evidence from excluded studies

Given the weak quality of evidence, the additional consideration of indirect evidence from our excluded studies might help to identify potential implications for further research. We excluded trials because (a) studies had no parallel group control or (b) patients had no significant or severe functional impairment at baseline (more than 50 % of the sample presented no or minor impairment).

(a) Excluded studies; no parallel group control

1. Educating clinicians in guideline-concordant glycemic control (HbA1c < 8 %) resulted in (1) higher use of insulin and oral anti-hyperglycemic medication, (2) lower mean HbA1c levels and fewer participants with inappropriate HbA1c level and (3) reduced episodes of hyperglycemia in a sample of 1,012 diabetic frail older adults [1]. We excluded this trial, because it used a historical control group design.

(b) Excluded studies; participants had no significant or severe functional impairment

1. A Swedish large-scaled registry study with 79,159 older individuals with T2DM, a mean age of 80.8 years and minor functional impairment revealed that the risk of hip fracture was higher in patients treated with insulin compared to patients with oral antidiabetics. The risk of non-skeletal fall injury did not differ among older persons with any DM medication but was consistently increased compared to older persons without DM [6].
2. Insulin therapy was a significant predictor of decline in instrumental activities of daily living at six years after baseline among 317 older persons with DM and minor physical impairment at baseline recruited in the Japanese Elderly Diabetes Intervention Trial (J-EDIT) [2].
3. Insulin use compared with non-use was significantly associated with reduced health related quality of life (SF-12, physical component summary) among 1,826 Chinese diabetic patients with initial stages of frailty managed in a public primary care setting [7].
4. A multicenter RCT compared the effects of vildagliptin versus placebo to reach individually defined HbA1c targets in 278 diabetic older persons (mean age 75) 24 weeks from baseline. Physical function was not measured and only a very small subgroup (< 10%) was classified as

frail. Although vildagliptin showed a significant effect on reaching the defined HbA1c target in non-frail persons, this effect was not found in frail older persons [4].

5. In a cohort study with 236 diabetic older adults (median age 69.5 years), 25% of subjects were frail, 72% pre-frail and 3% non-frail. Metformin users (n=175, 12 years median time of use) compared to non-users had a lower risk for frailty and presented fewer comorbidities [5].

Schlender et al. [3] concluded that the efficacy and safety of metformin use is similar or better than other specific or non-specific active treatments based on a review of 4 interventional and 11 mainly retrospective observational studies in older adults. However, the evidence was low and no studies reported on the frailty level of the participants.

References:

1. Lee SJ, Boscardin WJ, Stijacic Cenzer I et al. (2011) The risks and benefits of implementing glycemic control guidelines in frail older adults with diabetes mellitus. Journal of the American Geriatrics Society 59:666-672
2. Sakurai T, Iimuro S, Sakamaki K et al. (2012) Risk factors for a 6-year decline in physical disability and functional limitations among elderly people with type 2 diabetes in the Japanese Elderly Diabetes Intervention Trial. Geriatrics & gerontology international 12 Suppl 1:117-126
3. Schlender L, Martinez YV, Adeniji C et al. (2017) Efficacy and safety of metformin in the management of type 2 diabetes mellitus in older adults: a systematic review for the development of recommendations to reduce potentially inappropriate prescribing. BMC geriatrics 17:227
4. Strain WD, Lukashevich V, Kothny W et al. (2013) Individualised treatment targets for elderly patients with type 2 diabetes using vildagliptin add-on or lone therapy (INTERVAL): a 24 week, randomised, double-blind, placebo-controlled study. Lancet 382:409-416
5. Sumantri S, Setiati S, Purnamasari D et al. (2014) Relationship between metformin and frailty syndrome in elderly people with type 2 diabetes. Acta medica Indonesiana 46:183-188
6. Wallander M, Axelsson KF, Nilsson AG et al. (2017) Type 2 Diabetes and Risk of Hip Fractures and Non-Skeletal Fall Injuries in the Elderly: A Study From the Fractures and Fall Injuries in the Elderly Cohort (FRAILCO). Journal of bone and mineral research : the official journal of the American Society for Bone and Mineral Research 32:449-460
7. Wan EY, Fung CS, Choi EP et al. (2016) Main predictors in health-related quality of life in Chinese patients with type 2 diabetes mellitus. Quality of life research : an international journal of quality of life aspects of treatment, care and rehabilitation 25:2957-2965
